# Prediction of progression in pTa and pT1 bladder carcinomas with p53, p16 and pRb

**DOI:** 10.1038/sj.bjc.6601954

**Published:** 2004-06-29

**Authors:** A W Hitchings, M Kumar, S Jordan, V Nargund, J Martin, D M Berney

**Affiliations:** 1 Department of Histopathology and Morbid Anatomy, Bart's & The London School of Medicine & Dentistry, St Bartholomew's Hospital, West Smithfield, London EC1A 7BE, UK; 2 Department of Surgery, Bart's & The London School of Medicine & Dentistry, St Bartholomew's Hospital, West Smithfield, London EC1A 7BE, UK

**Keywords:** transitional cell carcinoma, immunohistochemistry, molecular markers, prognosis

## Abstract

Currently available prognostic tools appear unable to adequately predict recurrence and progression in non muscle-invasive bladder carcinomas. We aimed to assess the prognostic value of immunohistochemical evaluation of the cell cycle markers p53, p16 and pRb. Paraffin blocks were obtained from 78 cases of pTa and pT1 transitional cell carcinomas, for which long-term follow-up was available. Representative sections were stained using antibodies against p53, p16 and pRb. Altered marker expression was found in 45, 17 and 30% of cases, respectively. Concurrent alteration of two or three markers occurred in 19% of cases, and was significantly associated with grade and stage. In univariate survival analysis, the concurrent alteration of any two markers was significantly associated with progression. The greatest risk was produced by alteration of both p53 and p16, which increased the risk of progression by 14.45 times (95% confidence interval (CI) 3.10–67.35). After adjusting for grade and stage, this risk was 7.73 (CI 1.13–52.70). The markers did not generally predict tumour recurrence, except in the 25 pT1 tumours. In these, p16 alteration was associated with a univariate risk of 2.83 (CI 1.01–7.91), and concurrent p53 and p16 alteration with a risk of 9.29 (CI 1.24–69.50). Overall, we conclude that the immunohistochemical evaluation of p53 and p16 may have independent prognostic value for disease progression, and may help guide management decisions in these tumours.

The clinical course of non muscle-invasive transitional cell carcinomas of the bladder is unpredictable. Following primary resection, most patients with pTa or pT1 tumours will develop recurrences, while around 10–20% will suffer progression to potentially life-threatening muscle-invasive disease. Conventional prognostic variables such as grade and stage are unable to adequately predict which patients will suffer recurrence and progression, and this leads to difficulty in selecting the most appropriate management. The identification of a subset of patients requiring aggressive but curative management, such as cystectomy, would be a great advantage. Consequently, much attention has been focused towards the identification of prognostic markers of progression and recurrence.

In particular, promise has been shown in the evaluation of markers associated with the G1/S cell cycle checkpoint. The proteins p53, p16 and pRb are key components of this checkpoint, and their examination by immunohistochemistry is a valuable method for the molecular phenotyping of tumours. In this, altered expression patterns are used as surrogate indicators of underlying gene abnormalities. Absence of p16 and pRb expression is a sensitive means for identifying the statuses of the *INK4A* and *RB* genes, respectively (Zhang *et al*, 1994; Gorgoulis *et al*, 1998; Benedict *et al*, 1999; Orlow *et al*, 1999). In addition, recent work has indicated that elevated expression of pRb may represent upstream changes leading to pRb phosphorylation and inactivation, and should also be considered abnormal (Cote *et al*, 1998; Grossman *et al*, 1998). Mutation of the *TP53* gene results in the production of an abnormal p53 protein with a prolonged half-life (Finlay *et al*, 1988), which accumulates to concentrations detectable by immunohistochemistry.

Numerous studies in bladder cancer have established links between the expression of individual molecular markers and clinical outcome. However, it has become clear that single markers provide insufficient predictive power upon which to base management decisions for individual patients (Knowles, 2001). Several recent studies have indicated that evaluation of both p53 and pRb in bladder cancer provides greater predictive power than can be obtained from either marker alone (Cordon-Cardo *et al*, 1997; Cote *et al*, 1998; Grossman *et al*, 1998). Similarly, work by Korkolopoulou *et al* (2000) found that together p16 and p53 were predictive of overall survival in invasive bladder tumours, whereas individually they were not. In this study we provide, to our knowledge, the first thorough assessment of p53, p16 and pRb for their combined prognostic value in a well-characterised cohort of non muscle-invasive transitional cell carcinomas of the bladder.

## MATERIALS AND METHODS

A total of 121 patients were treated for primary non muscle-invasive transitional cell carcinoma of the bladder at The Royal London and St Bartholomew's Hospitals between 1982 and 1986. The study population was selected retrospectively from this group on the basis that (a) original tumour material was available and (b) satisfactory follow-up data could be obtained. No other exclusion criteria were made. The resulting cohort comprised 78 patients of whom 58 (74%) were male. The median age at diagnosis was 66 years (range 24–90), and the median duration of follow-up was 8.0 years (range 3 months–18.2 years). All haematoxylin–eosin (H&E) sections were examined by a histopathologist (DMB) to determine grade and stage according to the WHO (Mostofi *et al*, 1973) and TNM (Sobin and Wittekind, 2002) classifications, respectively. Representative slides were selected to perform immunohistochemistry on the corresponding paraffin block.

All patients were treated by transurethral resection with curative intent. None received intravesical BCG (bacillus Calmette–Guerin) for primary disease. Follow-up cystoscopies were performed on all cases initially at 3-monthly intervals, subsequently increased to 6-monthly intervals according to individual tumour characteristics. Recurrent disease was treated by transurethral resection/diathermy, with or without intravesical instillation of BCG.

Definitions for recurrence and progression were set prior to analysis of the data. Specifically, recurrence was defined as the reappearance of a biopsy-proven lesion. Progression was defined as advancement in stage or dedifferentiation to grade 3 disease (Santos *et al*, 2003).

Paraffin sections were cut at 3 *μ*m. Immunohistochemistry was performed using a standard avidin–biotin complex (ABC) method using the Vectastain Universal ABC Elite Kit (Vector Laboratories, Burlingame, USA). The primary antibodies were p16 (Novocastra Laboratories Ltd, Newcastle upon Tyne, clone 6H12, dilution 1 : 50), pRb (Novocastra Laboratories Ltd, clone 13A10, dilution 1 : 400), and p53 (DakoCytomation Ltd, Cambridgeshire, clone DO-7, dilution 1 : 100). Sections for p16 and pRb immunohistochemistry were heated for 4 min in 0.01 M citrate buffer (pH 6.0). For p53, antigen retrieval was by overnight incubation in 0.01 M citrate buffer at 60°C. Sections were counterstained with Gill's haematoxylin.

Negative and positive controls were included with each batch. For negative controls, duplicate sections for each case were stained by an identical method, except bovine serum albumin with azide was substituted for the primary antibody solution. Positive controls included a high-grade invasive breast carcinoma for p53, cervical intraepithelial neoplasia for p16 and normal tonsil for pRb.

Immunohistochemical evaluation was carried out qualitatively, by two investigators (DMB and AWH) at a double-headed microscope, who reached consensus for the percentage of positive tumour cells in each case. Only nuclear staining was considered specific for p53 and pRb, and all reactive nuclei were considered positive irrespective of intensity. For p16, both nuclear and cytoplasmic staining were included. Evaluation was performed blind, without knowledge of patient outcome or other clinicopathological variables.

Threshold percentages were applied to distinguish between normal and altered expression patterns, which were established prior to statistical analysis of the data. Specifically, p53 expression was defined as altered when at least 20% of cells showed nuclear staining (Serth *et al*, 1995; Lacombe *et al*, 1996; Cordon-Cardo *et al*, 1997; Sgambato *et al*, 1999), and p16 was defined as altered if less than 10% of cells were positive. For pRb, three categories were defined, as described by Cote *et al* (1998), namely absent (0%), normal heterogeneous (1–50%) and elevated (>50%). On the basis that absent and elevated expression were both altered patterns, these two groups were then combined.

Associations between marker alteration and grade and stage were examined using Fisher's exact test and the χ^2^-test for trend. Survival curves for progression and recurrence were obtained by the Kaplan–Meier method, and tested for equivalence by the log-rank test. Risk ratios and their 95% confidence intervals were calculated by Cox proportional hazards regression. Results were considered significant if the two-sided *P*-value was <0.05. Calculations were performed using SPSS (release 10.0.5, SPSS Inc, Chicago, USA). Ethical approval for this study was obtained from East London and City Health Authority Research Ethics Committee.

## RESULTS

**Figure 1:**
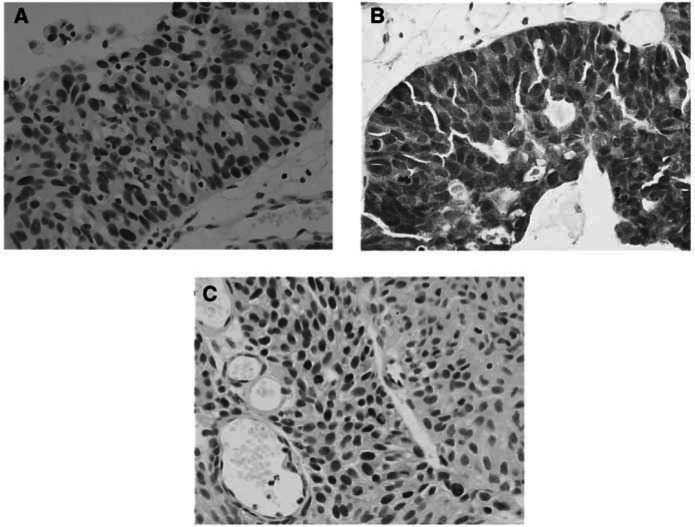
(**A**) Nuclear staining for p53. (**B**) Strong immunohistochemical staining for p16. (**C**) Nuclear staining for pRb.

For all three antibodies, the pattern of staining was frequently heterogeneous within a given tumour, with variation in both the percentage of positive cells and the intensity of staining (Figure 1

). Usually, the distribution of stained cells appeared random, although in 19/78 cases (24%) p16 appeared to preferentially stain the basal cell layers. All negative controls were reviewed for nonspecific staining, and all positive controls were reviewed to verify the adequacy of antigen-specific staining.

Cases were classified as normal or altered with respect to their immunophenotype for each marker. The distribution of cases between these categories is given in Table 1

**Table 1:** Molecular marker status grouped by grade and stage

		**Grade**		**Stage**	
**Molecular marker status**	***n***	**G1/2**	**G3**	**pTa**	**pT1**
p53		*P*=0.026		*P*=0.007	
Normal	43 (55%)	38 (62%)	5 (29%)	35 (66%)	8 (32%)
Altered	35 (45%)	23 (38%)	12 (71%)	18 (34%)	17 (68%)
					
p16		*P*=0.143		*P*=0.103	
Normal	65 (83%)	53 (87%)	12 (71%)	47 (89%)	18 (72%)
Altered	13 (17%)	8 (13%)	5 (29%)	6 (11%)	7 (28%)
					
pRb		*P*=0.130		*P*=0.793	
Normal	55 (71%)	46 (75%)	9 (53%)	38 (72%)	17 (68%)
Altered	23 (29%)	15 (25%)	8 (47%)	15 (28%)	8 (32%)

*P*-values obtained by Fisher's exact test.

, which also shows their relationship with grade and stage. p53 alteration occurred more frequently in grade 3 and stage pT1 disease (*P*=0.026 and 0.007, respectively, Fisher's exact test). No statistically significant associations existed between the markers themselves.

Cases were then further classified according to the number of markers with altered expression patterns as (a) all expressed normally, (b) one marker altered, or (c) two or three markers altered. Normal expression of all three markers was found in 25/78 cases (32%), while 15/78 tumours (19%) showed concurrent alteration of two or three markers. This index was strongly associated with both grade and stage (Table 2

**Table 2:** Combined marker status grouped by grade and stage

		**Grade**		**Stage**	
**Combined marker status**	***n***	**G1/2**	**G3**	**pTa**	**pT1**
		*P*<0.001		*P*=0.005	
All normal	25 (32%)	22 (36%)	3 (18%)	21 (40%)	4 (16%)
One altered	38 (49%)	34 (56%)	4 (24%)	26 (49%)	12 (48%)
⩾Two altered	15 (19%)	5 (8%)	10 (59%)	6 (11%)	9 (36%)

*P*-values obtained by χ^2^-test for trend.

).

### Predictors of progression

Progression to a more aggressive tumour phenotype occurred in 17/78 patients (21.8%) in this series. Grade 3 disease, stage pT1, and altered p16 expression were significantly associated with progression (*P*=0.003, 0.036, and 0.033, respectively, log-rank test). Altered p53 expression closely approached significance (*P*=0.064), while pRb alteration was not significant (*P*=0.150). Cox regression was used to calculate univariate relative risks for each of these variables. The greatest risk was conferred by grade 3 disease, which was associated with a relative risk of 3.88 (95% CI 1.50–10.09) compared to G1/G2 tumours. Stage pT1 increased the risk of progression by 2.66 times (CI 1.03–6.91) compared to pTa tumours. The relative risk for progression associated with altered p53 expression was 2.48 (CI 0.92–6.74), for p16 was 2.98 (CI 1.04–8.53) and for pRb was 2.01 (CI 0.76–5.28).

Overall, concurrent alteration of any two or three markers was strongly associated with progression, conferring a relative risk of 6.25 (CI 1.65–23.69, *P*=0.007) compared with a normal immunophenotype. To assess the risk associated with specific combinations of marker alterations, relative risks were calculated for each of the three marker pairings. The greatest risk for progression was produced by concurrent alteration of p53 and p16 (Table 3

**Table 3:** Relative risks for progression associated with combined marker status

**Variable**	***n***	**Relative risk**	**95% Confidence interval**	***P***
p53/p16				
Both normal	*35*	1.00		
One altered	*38*	2.42	0.76–7.74	0.136
Both altered	*5*	14.45	3.10–67.35	0.001
				
p53/pRb				
Both normal	*32*	1.00		
One altered	*34*	1.88	0.56–6.25	0.304
Both altered	*12*	4.77	1.27–17.84	0.020
				
p16/pRb				
Both normal	*46*	1.00		
One altered	*28*	2.16	0.78–5.97	0.138
Both altered	*4*	8.98	1.81–44.44	0.007

**Figure 2:**
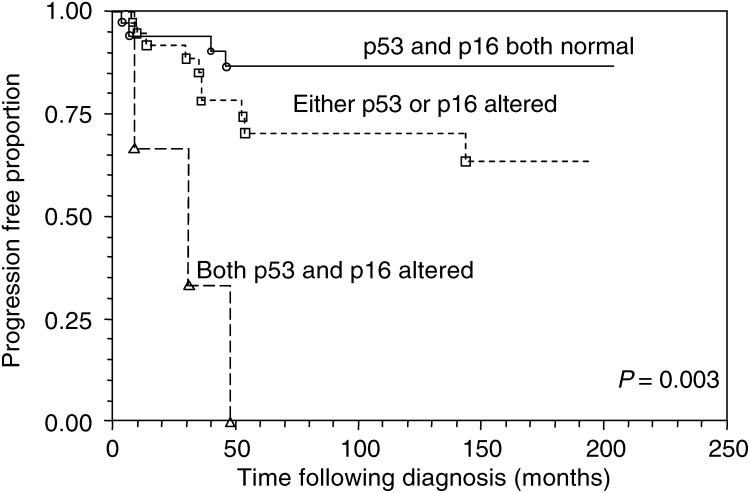
Kaplan–Meier curve for progression-free survival according to p53 and p16 status (*P*-value from log-rank test for trend).

Cox proportional hazards regression.

). The five cases with this immunophenotype had a relative risk for progression of 14.45 (CI 3.10–67.35) compared with tumours in which both markers were expressed normally. Of the 35 cases with entirely normal p53/p16 expression, four (11.4%) nevertheless progressed to a more aggressive phenotype. The progression-free survival according to p53/p16 status is illustrated in Figure 2

. Only three cases showed concurrent alteration to all three markers, and accordingly these were not examined as a separate group.

By entering the p53/p16 index with grade and stage in a Cox regression model, adjusted risk ratios were obtained (Table 4

**Table 4:** Adjusted risks for progression

**Variable**	**Relative risk**	**95% Confidence interval**	***P***
p53/p16			
Both normal	1.00		
One altered	1.77	0.44–7.14	0.420
Both altered	7.73	1.13–52.70	0.037
			
Grade and stage index			
G1/2, pTa	1.00		
G3, pTa or G1/2, pT1	1.13	0.25–5.16	0.876
G3, pT1	2.29	0.60–8.74	0.225

Cox proportional hazards regression.

). The concurrent alteration of both markers produced an adjusted risk ratio for progression of 7.73 (CI 1.13–52.70). The grade and stage index did not have independent prognostic value for progression within this model.

To assess whether the p53/p16 index retained independent prognostic value in the presence of the alternative pairings of p53/pRb and p16/pRb, these variables were added to the above model. In this case, no variable achieved significance. The p53/p16 index was closest to the significance level, with concurrent alteration to the two markers associated with relative risk of 6.44 (CI 0.71–58.85). By contrast, the risk was 1.76 (CI 0.27–11.66) for concurrent alteration to p53 and pRb, and 2.44 (CI 0.30–19.94) for p16 and pRb. G3pT1 tumours were associated with a risk for progression of 1.67 (CI 0.39–7.08) in this model.

When analysis was restricted to the 53 pTa tumours, none of the individual markers achieved statistical significance for the prediction of subsequent tumour progression. The univariate risks were 2.66 (CI 0.66–10.77) in the case of p53 alteration, 3.37 (CI 0.67–16.82) for p16 alteration, and 1.83 (CI 0.44–7.69) for pRb alteration. Of note, however, was the prognostic information provided by the combined p53/p16 index. This was associated with a relative risk of 2.67 (CI 0.59–12.01) for alteration to either p53 or p16, and of 15.27 (CI 1.41–165.59) for concurrent alteration to both markers. On multivariate analysis using a model containing the p53/p16 index and grade, no variable achieved significance for independent prognostic value.

The markers were not significant prognostic factors in the 25 pT1 tumours, for which the individual relative risks were 0.95 (CI 0.19–4.76) for p53 alteration, 2.34 (CI 0.55–10.03) for p16, and 1.90 (CI 0.51–7.09) for pRb. The p16/pRb index was the closest to significance in this cohort, being associated with a relative risk of 1.23 (CI 0.27–5.57) for alteration to either marker, and 5.28 (CI 0.88–31.72) for concurrent alteration to both markers.

### Predictors of recurrence

Recurrence occurred in 50/78 patients (64.1%) in this cohort. Cox regression analysis indicated that neither grade nor stage significantly predicted recurrence. Grade 3 disease conferred a relative risk of 1.28 (CI 0.67–2.45) compared to G1/G2 tumours, while the risk associated with stage pT1 *vs* pTa was 1.18 (CI 0.66–2.12). Similarly, analysis of individual molecular markers revealed no significant differences in recurrence rates between the normal and altered expression patterns. The relative risk for recurrence associated with p53 alteration was 0.65 (CI 0.36–1.15), for p16 was 1.54 (CI 0.74–3.19), and for pRb was 1.39 (CI 0.77–2.53).

On examining the molecular markers in pairs, only the combined p53/p16 status yielded differences in recurrence rates approaching statistical significance. A single alteration to either p53 or p16 appeared protective against recurrence, conferring a relative risk of 0.56 (CI 0.31–1.00) compared with a normal immunophenotype. Concurrent alteration to both markers, however, produced a 2.81-fold increase in the risk of recurrence (CI 0.94–8.40). After adjusting for grade and stage, the risk ratios for recurrence were 0.45 (CI 0.23–0.88) for a single alteration, and 2.21 (CI 0.68–7.19) for alteration to both markers. No other marker combination was able to improve on the predictive power of this model.

On confining the analysis to the 53 pTa tumours, no significant prognostic variables for recurrence were found. However, in the 25 pT1 tumours, p16 alteration was prognostic, increasing the risk of recurrence by 2.83 times (CI 1.01–7.91). Although p53 alteration was not individually associated with recurrence, the combination of p53 and p16 alteration was more informative. This index was associated with a risk ratio of 0.52 (CI 0.14–1.91) if one marker was altered, and 9.29 (CI 1.24–69.50) if both markers were altered. It was not possible to assess this risk satisfactorily against grade in multivariate analysis due to the absence of G1 tumours within this cohort. The p53/pRb and p16/pRb indices were not significant predictors of recurrence in this group.

## DISCUSSION

The findings of this study indicate that immunohistochemical evaluation of p53 and p16 may identify a subset of patients at high risk for progression to more aggressive disease. This may be of use in selecting patients for early aggressive therapy. In contrast, tumour recurrence is not generally predicted by these markers.

p53 expression was altered in 45% of cases, compared with 13–54% reported for pTa and pT1 bladder cancers in other series (Cordon-Cardo *et al*, 1997; Cote *et al*, 1998; Pfister *et al*, 1999; Sgambato *et al*, 1999; Korkolopoulou *et al*, 2000). Although high concordances have been demonstrated between *TP53* mutation and p53 expression (Esrig *et al*, 1993; Cordon-Cardo *et al*, 1994; Gorgoulis *et al*, 1998), wild-type p53 is also known to be expressed at detectable levels in some cases. However, given that this state also correlates with poor prognosis (Abdel-Fattah *et al*, 1998), its distinction is probably not important in practice. p53 alteration was associated with both grade and stage in this series, consistent with the view that it is a relatively late event in the evolution of the disease.

Wide variations are seen in the approaches used for evaluation of p16 staining. Loss of expression has been defined elsewhere as complete absence of staining in any part of the tumour (Geradts *et al*, 1995; Neihans *et al*, 1999; Santos *et al*, 2003), overall immunoreactivity of <5% of nuclei (Friedrich *et al*, 2001; Yang *et al*, 2002), and staining of <50% of nuclei in any part of the tumour (Korkolopoulou *et al*, 2000). Many studies disregard cytoplasmic staining on the assumption that it is nonspecific, although Benedict *et al* (1999) have indicated that it is p16-specific. In our series, the most common pattern was to see both cytoplasmic and nuclear staining, and in view of the practical difficulty of dense cytoplasmic staining making assessment of nuclear staining unreliable, both were counted as positive. Altered expression was defined as overall positivity of <10% of cells. On this basis, we identified 13/78 cases as p16-altered (17%), which is comparable with the 17–25% reported for bladder tumours in other series (Neihans *et al*, 1999; Korkolopoulou *et al*, 2000; Friedrich *et al*, 2001).

Recent evidence has indicated that for pRb, both absent (0%) and elevated (>50%) expression should be considered abnormal (Cote *et al*, 1998). Using these criteria, we found 30% of cases to be altered with respect to pRb expression, compared to the higher figure of 55% reported by Cote *et al* (1998).

Our findings provide further evidence for the value of phenotype-associated marker panels over single molecules in predicting progression in pTa and pT1 bladder cancers. On univariate analysis, all three markers appeared to be related to progression, but only p16 alteration was significant in this respect. p53 alteration closely approached, but did not reach, significance in this cohort. This result is similar to that of Grossman *et al* (1998) but contrasts with others (Serth *et al*, 1995; Cordon-Cardo *et al*, 1997), reflecting the sometimes inconsistent relationship seen between p53 and disease progression in different study cohorts. However, the concurrent alteration of any two markers was significantly associated with progression, and conferred a greater risk than that associated with any marker alone. The pairing of p53 and p16 appeared most useful in this respect, with concurrent alteration to these two markers conferring almost an eight-fold increase in the likelihood of disease progression after adjustment for grade and stage. The index did not provide independent prognostic information when the alternative pairings of p53/pRb and p16/pRb were also included in the model, which perhaps reflects the inherent overlap in the information provided by the three marker indices.

There remains a need for an improved negative prognostic tool in these tumours. In this series, however, four of the 35 cases with normal p53 and p16 expression progressed to a more aggressive phenotype. Thus, while these markers may have a role in identifying patients at high risk of progression, it would appear that they are less suitable for identifying low-risk patients.

In the 53 pTa tumours, the p53/p16 combination remained the most informative predictive variable on univariate analysis, although failed to yield significant prognostic information when assessed against grade in multivariate analysis. The need for improved prognostic variables is arguably greater in pT1 tumours, and on analysis of the 25 pT1 tumours in this series the markers failed to demonstrate significant prognostic value. Nevertheless, there remained a trend towards a greater risk in tumours with concurrent alteration to two markers as opposed to those with single marker alteration, with the p16/pRb index most closely approaching significance in this respect. We speculate that in a larger cohort this trend might become significant.

Escape from G1/S checkpoint control is important in malignant progression, linked with increased proliferative and decreased apoptotic capacity. Given the complexity of the molecular pathways regulating the checkpoint, it seems likely that various degrees of dysfunction could result from different combinations of molecular abnormalities. p53, p16 and pRb are key components in this checkpoint, and as such, their inactivation disrupts its normal function. By removing collateral and ‘fail-safe’ pathways, the inactivation of more than one of these molecules might be expected to increase the severity of this dysfunction. This may account for the increased predictive power apparently offered by simultaneous examination of multiple markers in relation to disease progression.

In contrast, recurrence was not generally associated with the status of these markers. An interesting exception to this was in the 25 pT1 tumours, in which p16 alteration was prognostic, and further improved by combined analysis with p53. It would be unwise to draw conclusions on this from such a small cohort, however, it is an intriguing point worthy of further exploration. Other series have found the markers to be largely nonprognostic in relation to tumour recurrence (Pfister *et al*, 1999; Korkolopoulou *et al*, 2000; Friedrich *et al*, 2001) and, taken together, these findings remain broadly consistent with the view that recurrence is dictated by factors less amenable to prediction by cell cycle markers. Recurrence may occur by cell seeding from an initial tumour, or separately from different areas of dysplastic epithelium.

Certain limitations must be borne in mind when drawing conclusions from these data. Firstly, the evaluation of staining in this study was qualitative. However, it represents the most likely method by which such investigations would be interpreted in routine clinical practice. In this respect, it may have advantages over formal quantification methods which, although possibly less subjective, are time-consuming and would probably be impractical outside a research setting.

Secondly, care must be taken in drawing comparisons with other studies. In addition to the different approaches for evaluation of staining mentioned above, differences also exist in the immunohistochemistry protocols used between laboratories. This can lead to variability in results (Fisher *et al*, 1994; McShane *et al*, 2000). The choice of antibody is particularly relevant. Masters *et al* (2003) suggest that the DO-7 anti-p53 antibody (Dako), used in the present study, is less strongly associated with pTa/pT1 bladder tumour progression than the alternative pAb1801 antibody (Novocastra). However, they also report the presence of pale but reproducible staining of some tumours with DO-7, which was not considered positive for the purpose of analysis, but noted as meriting further investigation. All intensities of staining were considered positive in the present study, and we therefore possibly provide evidence for the value of including such tumours in survival analyses.

Thirdly, the definition used for bladder tumour progression is sometimes variable. Most studies define progression as advancement to muscle-invasive disease. However, given that grade 3 and stage pT1 tumours represent more malignant disease requiring more aggressive treatment, we set a wider definition to include progression to such phenotypes. While we believe that this definition is more applicable to clinical practice than the relatively narrow definition used elsewhere, the discrepancy must be borne in mind when comparisons are being drawn.

In spite of these caveats, our findings indicate that patients with concurrent alteration to both p53 and p16 were at a significantly higher risk of disease progression than patients with a normal immunophenotype for these markers, after adjusting for grade and stage. To our knowledge, this is the first report of this effect. Moreover, we are not aware of any other study in which p53, p16 and pRb expression has been characterised in the same cohort of pTa and pT1 bladder cancers, and assessed collectively in relation to disease progression. In view of this, it is noteworthy that the concurrent alteration of p53 and p16 appeared more informative than alteration of p53 and pRb, for which prognostic value has already been established (Cordon-Cardo *et al*, 1997; Cote *et al*, 1998; Grossman *et al*, 1998). Given the significantly increased risk for progression associated with p53 and p16-altered nonmuscle-invasive bladder cancers, patients presenting with such tumours may be better served by early treatments such as immunotherapy, chemotherapy or even cystectomy.
